# The Indian Bidi Industry: Trends in Employment and Wage Differentials

**DOI:** 10.3389/fpubh.2020.572638

**Published:** 2020-10-07

**Authors:** Monika Arora, Pritam Datta, Avnika Barman, Praveen Sinha, Vineet Gill Munish, Deepika Bahl, Soumyadeep Bhaumik, Gaurang P. Nazar, Fikru Tullu

**Affiliations:** ^1^Health Promotion Division, Public Health Foundation of India, Gurugram, India; ^2^Health Related Information Dissemination Amongst Youth (HRIDAY), New Delhi, India; ^3^National Institute of Public Finance and Policy, New Delhi, India; ^4^World Health Organization Country Office for India, New Delhi, India; ^5^The George Institute for Global Health, New Delhi, India

**Keywords:** employment, wages, bidi, India, tobacco

## Abstract

**Background:** The bidi industry in India is predominantly an unorganized sector. It continues to enjoy tax benefits, arguably, to protect bidi workers' interests and employment. Our objective was to study trends in employment and wage differentials in the bidi industry using nationally representative data.

**Methods:** We studied trends in employment and wages in the bidi industry using secondary data from the National Sample Survey Office (NSSO) and the Central Statistics Office (CSO), Government of India–the Annual Survey of Industries (ASI) (2000–2001 to 2011–2012) and Enterprises Survey (2000–2001, 2005–2006, 2010–2011).

**Results:** The bidi industry contributed to only 0.65% of the total gross value added (GVA) by the entire manufacturing industry. Employment in this industry was primarily through contractors. Bidi workers earned only 17% of wages compared to workers in other manufacturing industries. Although females constituted the majority of bidi workers, they earned INR 7,000 to 8,000 (USD 155.7 to 178) less than male bidi workers annually. Despite the increase in bidi industry profits from INR 1.7 billion (USD 37.8 million) in 2005–2006 to INR 12.8 billion (USD 285 million) in 2010–2011, the wages of bidi workers have continued to decline over this period.

**Conclusion:** Bidi workers earn much less compared to workers in other manufacturing industries and are subject to income inequality. There is a need to increase registration of the bidi industry for better administration of taxes and prevention of exploitation of the bidi workers. Skill building and alternative employment could provide better job quality, wages, social security and employment benefits.

## Introduction

Bidi (an indigenous smoking product made my rolling a dried, rectangular piece of tendu leaf with 0.15–0.25 grams of dried, flaked tobacco and secured with a thread) is the most common smoked tobacco product in India (1). About 7.7% of adults in India consume bidi (2), which has a market share of 85% of all smoking products (3, 4), Health consequences of bidi are not confined to its users only but extend to those involved in the bidi manufacturing process (5). In its initial phases, the bidi industry was largely confined to the organised sector but it gradually shifted to the unorganised sector. This shift was attributed to strict government rules, regulations and policies on the organised sector and the tax liberalisation in the unorganised sector (6). Currently, bidi manufacturing units operational in the country can be grouped into two categories viz. [a] registered companies and [b] unregistered companies (7). The registered companies are the large entities producing more than 2 million branded bidis per year, are bound to pay excise duty on their product and are legally bound to follow labour laws; while, the unregistered companies have been exempted from tax payments, as they manufacture <2 million bidis a year (8). However, with new GST policy in 2019, enterprises with a turnover of INR 4 million (USD 58114; 1USD = 68.83) have been exempted from the tax from the year 2019–2020 (9). With the introduction of this new norm, nearly 98% of the bidi manufacturing units would stay out of the tax bracket (9).

While bidi industries are observed in rural and semi-urban areas of India, variations exist in terms of the capital invested, size of bidi industry, employment size, gender, and child composition of workers (10). Also, within the industry, variations are observed in employment types i.e., full-time, part-time and the contractual workers. The bidi sector contributed around 0.9% of total employment in India in 2005–2006 (11). In the same year, 4.16 million workers were employed in the bidi industry and amongst them, 3.42 million workers were engaged in full-time work while 0.74 million workers were employed part-time (11). Women and children constitute 90% of its workforce and are mainly employed because of their proficiency in rolling bidis (10). Men are being employed in this industry but mainly in the factory system, whereas 90% of women are involved in the home-based system of bidi making (10).

As bidi industry continues to enjoy tax benefits, arguably, to protect bidi workers' interests and employment, it is essential to understand the overall employment structure of the industry and wages earned by the bidi industry workers in India.

## Materials and Methods

### Study Design and Data

We studied trends using secondary data to examine the contribution of the bidi industry in terms of employment, income, profit, and output of the industry. Estimates were based on data from the National Sample Survey Office (NSSO) and the Central Statistics Office (CSO), Ministry of Statistics and Programme Implementation (MOSPI), Government of India. Data sources included the Annual Survey of Industries (ASI) and Enterprises Survey. Ethical approval for this study and written informed consent from the participants of the study were not required in accordance with local legislation and national guidelines, as the study comprised of analysing secondary anonymous datasets.

### The Annual Survey of Industries (ASI)

The ASI is an annual survey conducted by the CSO to gather information on industrial activities of the registered manufacturing sector in India (12). It is the prime source of industrial statistics in India and provides statistical information to assess and evaluate, objectively and realistically, changes in growth, composition, and structure of the organised manufacturing sector. To make findings of this study comparable with the unorganized bidi manufacturing sector, the ASI unit level data were obtained for the year 2000–2001 to 2011–2012 (12).

#### Enterprises Survey

The Enterprises Survey is an important industrial data source for the unorganised manufacturing sector in India. Following the first Economic Census of 1977, small establishments and enterprises not employing any hired worker [henceforth called “own account enterprises” (OAEs)] engaged in manufacturing and repairing activities were surveyed on sample basis in the 33rd round of the National Sample Survey (NSS) during 1978–1979 and the latest survey conducted in 2010–2011 (NSS 67th round). Under the Enterprise Survey conducted by NSSO, a survey on unorganized manufacturing activities is carried out sequentially. It provides data on gross value added (GVA), employment and other variables for unregistered or unincorporated enterprises that are not covered under ASI. Gross value added (GVA) is defined as the value of output less the value of intermediate consumption. It is used to measure the output or contribution of a particular sector. In the present study, we used data from 56th (2000–2001) (13), 62nd (2005–2006) (14) and 67th (2010–2011) (14) NSS rounds.

### Measures

The study included variables such as profit margin, the share of profit in total output, annual wages, wage inequality by gender and wages by type of employment.

### Statistical Methods

We studied trends using the secondary data to measure the different dimensions of the bidi industry: (i) type of employment in bidi industry, (ii) profits earned by bidi industry over the time (iii) wages earned by the bidi workers in comparison to other manufacturing units (iv) existence of wage inequalities if any between direct vs. contractual workers, male vs. female workers and managerial and supervisory level staff vs. other bidi workers. All profit estimations were adjusted for inflation using the Wholesale Price Index [WPI] (MP) base: 2004–2005=100. These have been presented in terms of number of units, number of workers and wages of bidi workers. All the statistical analyses were performed using STATA v.13.1 (StataCorp, LP Texas).

## Results

The estimated number of bidi manufacturing units (Units refers to bidi manufacturing factories or enterprises) as a percentage share of the estimated number of total manufacturing units in 2010–2011 was apparently significant at 12.79%, in terms of GVA from bidi manufacturing. Bidi manufacturing contributed to only 0.65% of the total GVA contributed by the entire manufacturing sector, this equates to INR 48.2 billion (USD 1.1 billion; 1USD =44.95 for year 2004–2005 here on) (data not shown).

### Employment Provided by the Bidi Manufacturing Industry

In terms of providing employment, the bidi industry accounted for only 7.04% of the entire manufacturing industry. It has been estimated that in 2000–2001, there were 3.56 million workers involved in bidi manufacturing and this fell to 3.32 million in 2010–2011 (data not shown). The bidi industry employs different types of workers, the majority of whom are either directly employed by firms (owners or hired workers) or employed through contractors (indirect employees). In addition, the registered sector employs staff for managerial and supervisory purposes and also documents unpaid workers and other employees.

#### Relative Size of Employment Provided by the Registered and Unregistered Bidi Manufacturing Sector

The number of workers employed in the registered sector varied from 0.4 million in 2000–2001 and 2005–2006 to 0.3 million in 2010–2011. The unregistered sector numbers varied from 3.1 million workers in 2000–2001 to 4.1 million workers in 2005–2006 and 2.9 million workers in 2010–2011. Majority of employment in the bidi industry is provided by the unregistered sector, and this proportion (88.6, 91.1, and 90.6% for years 2000–2001, 2005–2006, and 2010–2011, respectively) has remained mostly constant over the years.

##### Employment in the registered bidi manufacturing sector by type of employment and employment size of enterprises

Table 1 provides estimates of the number of workers in registered bidi manufacturing, by type of employment. Overall, the total number of workers in registered bidi manufacturing has declined over the decade 2000–2001 to 2010–2011. The foremost observation being that total direct employment has steadily declined over the study period. Among directly employed workers, the number of female workers was more than double that of male workers in all three study years.

**Table 1:** Number of workers in registered bidi manufacturing, by type of employment.

**Year**	**Directly employed male workers**	**Directly employed female workers**	**Total directly employed workers**	**Workers employed through contractors**	**Total workers employed (Direct + Contractual)**	**Managerial and supervisory staff**	**Other employees**	**Unpaid family members/proprietor, etc**	**Total number of people engaged**
2000–2001	42,095	80,655	122,750	263,942	386,692	2,993	10,029	Not surveyed in this round	399,714
2005–2006	37,565	75,103	112,668	299,744	412,412	3,908	9,249	2,100	427,670
2010–2011	29,326	68,627	97,953	257,910	355,863	2,879	6,542	1,659	366,944

Table 2 shows the number of workers in registered bidi manufacturing as a percentage share, classified by the employment size of units. The largest proportion of workers in registered bidi manufacturing was employed in factories that employed more than 500+ workers. The registered sector units employing 0–10 workers provided employment to only 0.96% of the total bidi manufacturing workforce in 2010–2011 (Table 2).

**Table 2:** Estimated percentage of registered bidi manufacturing workers (number of registered bidi manufacturing workers), by employment size of enterprises.

**Year**	**Estimated number of registered bidi manufacturing workers**							**Total No. of workers (in millions)**
	**0–10**	**11–20**	**21–50**	**51–100**	**101–200**	**201–500**	**500 +**	
2000–2001	0.03% (115)	0.09% (362)	0.51% (2,049)	0.91% (3,663)	1.90% (7,614)	5.71% (22,915)	90.84% (364,346)	0.401
2005–2006	0.08% (361)	0.27% (1,167)	1.18% (5,028)	2.00% (8,560)	3.55% (15,195)	11.37% (48,646)	81.54% (348,714)	0.427
2010–2011	0.96% (3,538)	1.53% (5,617)	2.36% (8,662)	4.86% (17,822)	4.42% (16,227)	6.15% (22,557)	79.72% (292,521)	0.367

##### Employment in the unregistered bidi manufacturing sector by type of employment and employment size of enterprises

Table 3 shows employment in the unregistered sector by the two types of enterprises, namely, OAMEs (Own Account Manufacturing Enterprises (OAME): A small manufacturing enterprise run by household members that do not hire any workers on a fairly regular basis) and Establishments: An enterprise which has at least one hired worker on a fairly regular basis) (15). The majority of workers were employed in OAMEs, i.e., they were self-employed. This showed that bidi manufacturing is largely concentrated in the unregistered sector, with the majority of workers rolling bidis themselves, therefore, making the industry largely self-employed and labor-intensive. Establishments, on the other hand, employed ~3% of workers in 2000–2001 (Table 3). This share fell to 1% in 2005–2006 and declined further to 0.7% in 2010–2011.

**Table 3:** Estimated percentage of unregistered bidi manufacturing workers (number of unregistered bidi manufacturing workers in millions), by type of enterprises.

**Year**	**OAME**	**Establishment**	**Total**
2000–2001	96.93% (3.06)	3.07% (0.1)	100% (3.16)
2005–2006	98.96% (4.11)	1.04% (0.04)	100% (4.15)
2010–2011	99.31% (2.94)	0.69% (0.02)	100% (2.96)

*OAME, Own Account Manufacturing Enterprises*.

Results in Table 4, provided a similar picture of employment in unregistered bidi manufacturing, except that the workers were classified simply as either being employed full-time or part-time. In OAMEs, there were more full-time workers. This was consistent, as OAMEs were self-owned manufacturing units. As in the registered sector, there were more female workers (total) in the unregistered sector. In fact, the total number of female workers (OAMEs + establishments) made up 82.84% (*n* = 2,448,388) of the total unregistered sector bidi manufacturing employment in 2010–2011. In OAMEs, in 2010–2011, female workers formed 82.17% (*n* = 2,084,618) of the total full-time workforce and 90.55% (*n* = 360,590) of the total part-time workforce.

**Table 4:** Number of workers in unregistered bidi manufacturing, by type of employment.

**Year**	**OAME**							**Establishment**							**Total workers employed**
	**Full-time**		**Total full time workers**	**Part-time**		**Total part time worker**	**Total workers**	**Full-time**		**Total full time worker**	**Part-time**		**Total part time worker**	**Total workers**	
	**Female**	**Male**		**Female**	**Male**			**Female**	**Male**		**Female**	**Male**			
2000–2001	1,817,788	753,593	2,571,381	394,235	94,343	488,578	3,059,959	20,191	66,744	86,935	4,752	5,084	9,836	96,770	3,156,729
2005–2006	2,586,262	791,770	3,378,032	555,050	180,114	735,164	4,113,195	14,847	22,677	37,524	4,395	1,357	5,752	43,276	4,156,472
2010–2011	2,084,618	452,328	2,536,946	360,590	37,619	398,209	2,935,154	2,849	16,318	19,167	331	827	1,158	20,325	2,955,479
Total workers employed	6,488,668	1,997,691	8,486,359	1,309,875	312,076	1,621,951	10,108,308	37,887	105,739	143,626	9,478	7,268	16,746	16,0371	10,268,680

*OAME, Own Account Manufacturing Enterprises*.

More full-time male workers were employed in unregistered establishments than females. In 2010–2011, female workers in unregistered establishments formed only 14.86% (*n* = 2,849) and 28.61% (*n* = 331) of the total full-time and part-time workforce, respectively. Overall, the findings showed consistently that the bidi manufacturing industry primarily works as small-sized units, where women are primarily engaged in bidi rolling.

#### Profit in the Registered Bidi Sector by Employment Size of Enterprise

In this study, profits for 2000–2001 were not estimated because of data issues. In addition, profit could not be calculated for (bidi) enterprises in the unregistered sector as it formed a part of the working owner's income, and was therefore, not distinguishable. The profit of the registered bidi sector, disaggregated by employment size of enterprise has been shown in Table 5. The total profit accrued by registered bidi manufacturing in 2005–2006 was INR1.7 billion (USD 37.8 million) which increased to INR12.8 billion (USD 285 million) in 2010–2011. While small-size units were the most abundant in the registered sector, the maximum number of workers were employed by large units employing 500+ workers per unit. In 2010–2011, most profit contributions were made by units that employed between 51 and 100 workers per unit (Table 5). Therefore, even though small-sized units were the most prevalent in the registered sector, they did not provide ample employment or generate significant economic value to the bidi manufacturing industry.

**Table 5:** Profit in registered bidi sector, disaggregated by employment size of enterprises.

**Employment size of enterprises**	**Profit, 2005–06 (INR million)**	**Profit, 2010–11 (INR million)**
0–10	34.08 (1.97%)	383.34 (2.99%)
11–20	3.49 (0.20%)	75.72 (0.59%)
21–50	60.23 (3.48%)	191.02 (1.49%)
51–100	48.68 (2.82%)	9618.17 (75.11%)
101–200	390.29 (22.58%)	31.92 (0.25%)
201–500	516.53 (29.89%)	414.85 (3.24%)
500+	675.06 (39.06%)	2090.40 (16.32%)
Total profit	1728.36	12805.41

*INR, Indian Rupees*.

*For the year 2005-2006, total profit INR 1728.36 ≈ USD 37.8 million*.

*For the year 2010-2011, total profit INR 12805.41 ≈ USD 285 million*.

**Figure 1:**
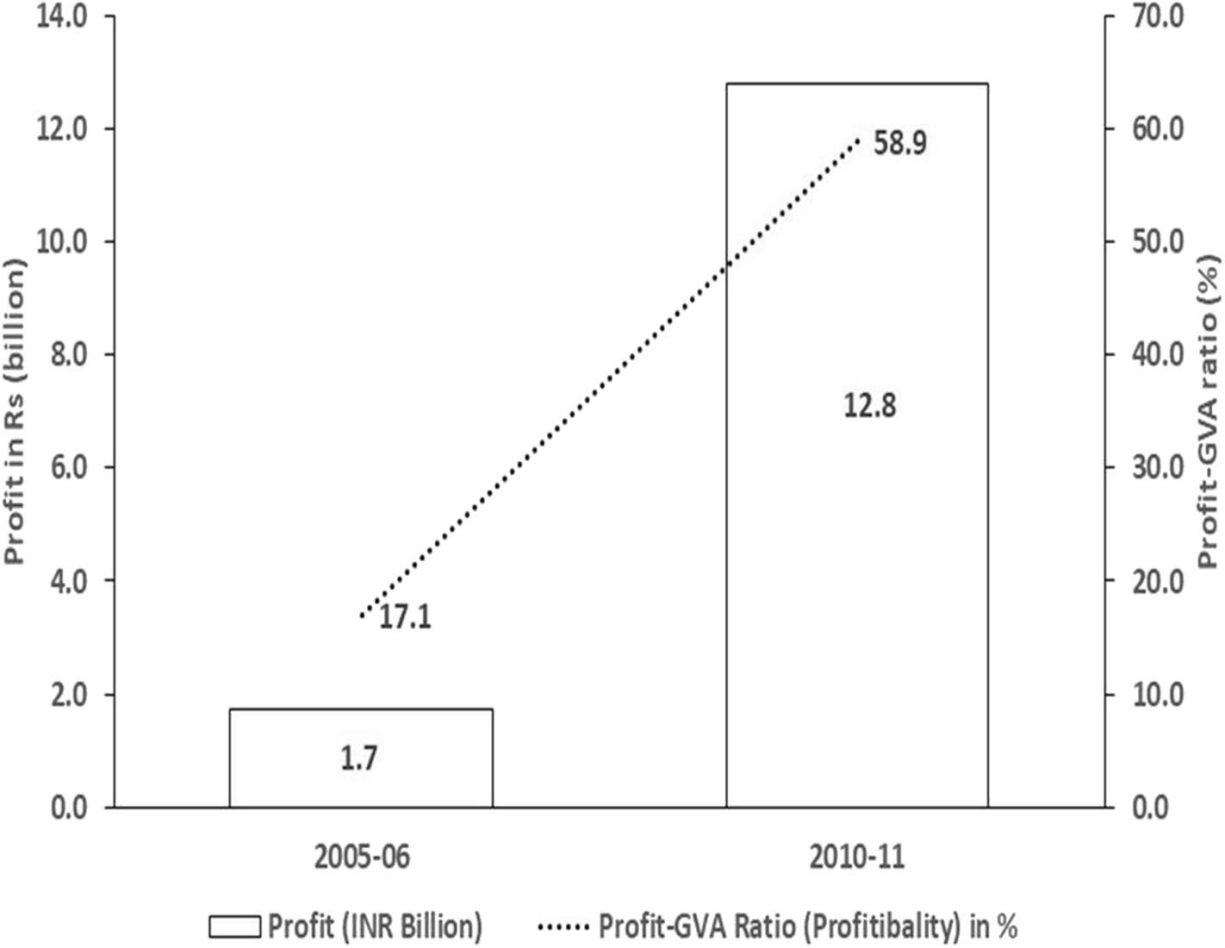
Profit and profit-GVA ratio in registered bidi manufacturing section.

Increase in profit, as well as the profit-GVA ratio, is shown in Figure 1. Profit-GVA ratio give a true picture of profits because it takes into account the output of the industry, thereby enabling year-to-year comparison (Figure 1). The profit-GVA ratio increased tremendously between 2005–2006 and 2010–2011 from 17.1 to 58.9%. In this study, all profit estimations have been adjusted for inflation using the WPI (MP) base: 2004–2005 = 100.

#### Wages of Workers in Registered Bidi Manufacturing Unit

An equity-focused analyses was conducted by analysing wages earned by bidi workers to be able to determine wage discrimination, if any, between direct vs. contractual workers, male vs. female workers and managerial and supervisory level staff vs. other bidi workers. Table 6 presents the average annual wage per worker earned in the registered bidi manufacturing sector by types of employment studied above.

**Table 6:** Annual average bidi wage per worker in the registered sector, by employment status [wage in Indian Rupees (INR)].

**Year**	**Directly employed male wages**	**Directly employed female wages**	**Directly employed total wages**	**Employed through contractor wages**	**Total wages (direct + contractual)**	**Managerial and supervisory staff wages**	**Other employees wages**	**All workers and employees**
2000–2001	23831.73	16094.04	18747.55	16289.38	17069.69	89969.24	39666.81	18182.57
2005–2006	20533.51	14205.69	16315.45	13347.97	14158.67	72561.77	35272.79	15153.94
2010–2011	22395.43	15784.71	17763.85	12010.85	13594.39	114462.88	43248.84	14920.52

*INR, Indian Rupees*.

Wages were segregated by employment type and also by gender. In 2010–2011, on average, bidi workers in the registered sector earned INR 14920.5 (USD 332) annually which was less than the annual average total wage earned by bidi workers in 2000–2001 (INR 18182.6; USD 404.5) (Table 6). When comparing these wages with workers engaged in other registered sectors, this amounted to only 17% of the annual average wage earned per worker in the registered manufacturing industry (Table 7).

**Table 7:** Wage inequality in different employee types in the registered bidi manufacturing sector (Percentage of wages earned by the employees in registered bidi manufacturing sector in comparison to other manufacturing sector).

**Year**	**Directly employed**	**Employed through contractors**	**Managerial and supervisory**	**Other Employees**	**All registered bidi sector workers**,
2000–2001	32%	51%	46%	43%	25%
2005–2006	28%	40%	29%	35%	20%
2010–2011	29%	29%	36%	38%	17%

Contrary to employment proportions, the average wage accrued by females was less than that of males. In 2000–2001, female workers earned INR 7737.7 (USD 172.1) less per year than males, on average; and in 2005–2006, the wage disparity between male and female workers was lower at INR 6327.8 (USD 140.8). In 2010–2011, however, not only did both male and female average annual wages increase, but the wage disparity increased with directly employed male workers earning INR 6610.7 (USD 147.1) more on average compared to female workers. Overall, the annual average wage earned per directly employed worker has been declining over the years, due to a drop in the total number of directly employed workers. On the other hand, the annual average wages earned by managerial workers were much higher compared to those of direct and contractual workers. In 2010–2011, the average annual wage for a managerial or supervisory staff was INR 11462.9 (USD 255) compared to INR 89969.2 in 2000–2001

Contractual workers accounted for the biggest share of total workers in the registered bidi manufacturing sector. However, the annual average wage earned by a contractual worker was lower than that earned by a directly employed worker. In fact, they earned the lowest average wage in registered bidi manufacturing (Table 6) compared to all other employees.

**Figure 2:**
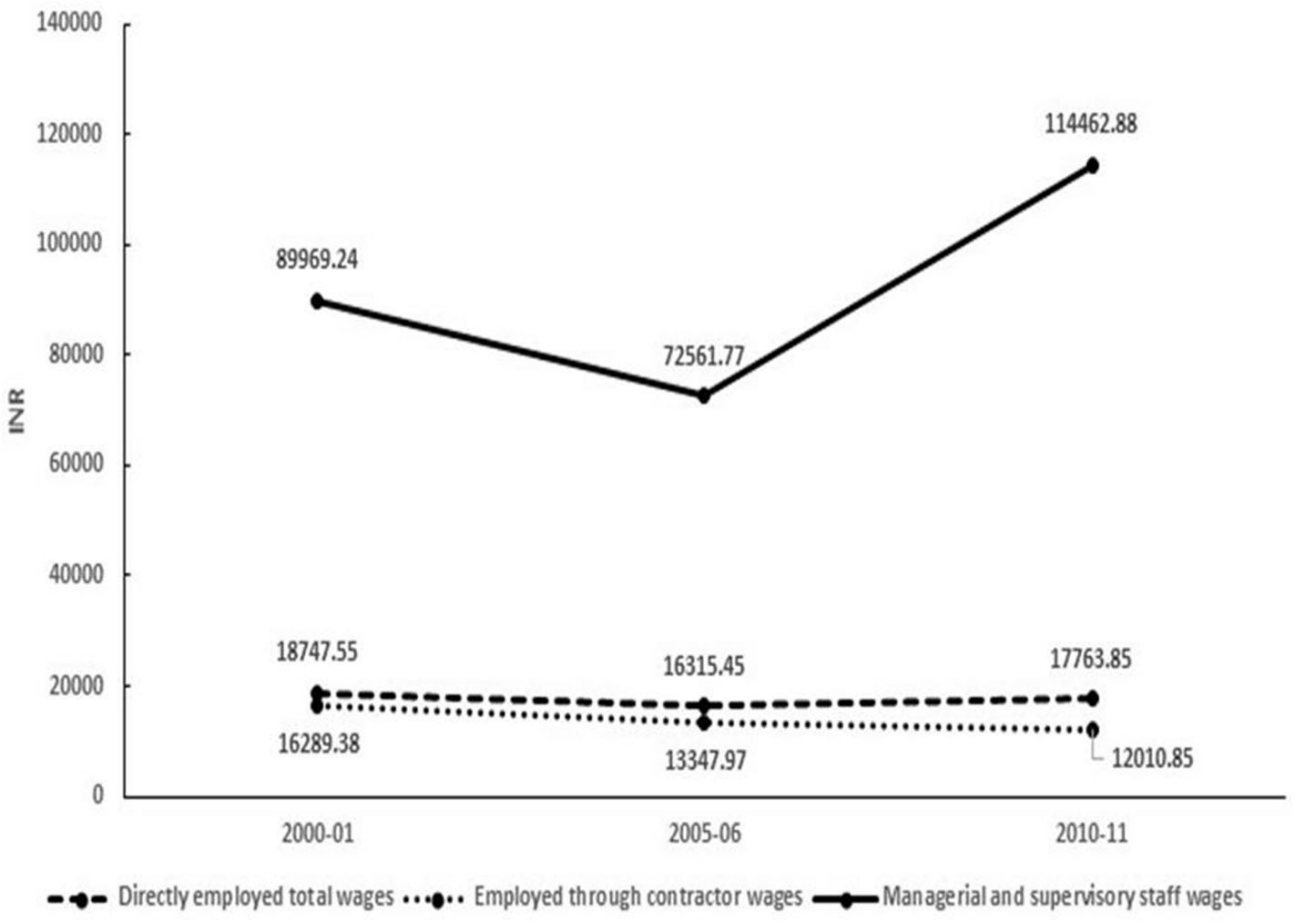
Annual average bidi wage per worker in the registered section, by employment status (wage in INR).

The average annual wages of directly employed as well as contractual workers decreased between the period 2000–2001 and 2005–2006. However, between 2005–2006 and 2010–2011, the wages of directly employed workers increased (but never to the levels of 2000–2001) and that of contractual workers kept on decreasing further. In contrast, for managers and supervisory level staff, although there was a slight dip in salary in 2005–2006, their salary rose in 2010–2011 and was much above the 2000–2001 levels (Figure 2).

We also attempted to estimate the ratio of the average annual wage of a worker in registered bidi manufacturing to the average annual wage of a worker in other registered manufacturing sectors. Table 7, shows that in 2000–2001, registered bidi workers earned 25% of wages earned by workers in in other manufacturing sector. Over the years, wages paid to bidi workers in the registered sector declined to 20% in 2005–2006 and 17% in 2010–2011.

## Discussion

In 2010–2011, in spite of the bidi industry employing 7.04% of workers in the entire manufacturing industry, its actual economic contribution was only a miniscule 0.65% of the total GVA of the entire manufacturing sector. This indicates that bidi manufacturing, overall, makes little contribution to the national economy, thus dispelling the myth of the “significant economic contribution” by the bidi industry as a reason for stalling stringent tobacco control measures. Further, the WHO recommends higher taxes on tobacco products as best practice for tobacco control (16). Moreover, previous Indian studies suggest that there is ample scope for increasing taxes on *bidi* products in India which can go up to 100% of the current level, without any loss of revenue to the government (17). However, in India, even post GST the tax burden on bidi is estimated to be 22% in contrast to 16% pre-GST; and unlike cigarettes and smokeless tobacco, there is no additional cess imposed on bidis (18).

Registration of all *bidi* manufacturing units is important in ensuring that *bidi* workers are not exploited. There is evidence of exploitation of *bidi* workers by middlemen, inadequate social security benefits provided to them and their families, lack of access to government programmes and growing poverty and vulnerability of families involved in the profession (19, 20). Given that the majority of *bidi* manufacturing units are in the unregistered sector, and almost all of the unregistered *bidi* manufacturing is comprised of OAMEs, it is evident that the majority of *bidi* sector is small-scale and unregistered in nature, with little employment benefits. The exploitation of *bidi* workers is more worrisome as wages over the years have in real terms remained stagnant and there is a move towards contractual employment and reduction in directly employed workers. Considering the employment provided by the *bidi* industry, it is evident that 89% of employment is in the unregistered sector. The segmented nature of the *bidi* sector is a major hurdle in registering, regulating and tracking these units by the Ministry of Labour and Employment and other authorities who are mandated to protect the rights and entitlements of *bidi* workers and prevent their exploitation. Also, over the years, the share of OAMEs is increasing, which does not benefit *bidi* workers. This is further complicated by the fact that there is subcontracting in the sector and this is largely done by the larger units, to probably avoid paying employment benefits to *bidi* workers, and to stay outside the tax system as per Indian laws and regulations. The analyses presented in this study indicate that even in the registered sector, more than half the employment in *bidi* manufacturing is through contractors. A move towards registration and decreased contractual employment will enable monitoring of worker benefits and wages to this large number of *bidi* workers and ensure they are not denied of their rights.

The registered *bidi* manufacturing units are shifting to enterprises employing between 0–10 and 11–20 workers. Therefore, over a period of 11 years, the average size of registered *bidi* manufacturing units, in terms of employment size has declined and more small-sized units have come into existence. A likely explanation for the preference of small-sized units (<20 workers) by the *bidi* industry could be to avoid providing benefits such as Employee's Provident Fund (EPF) to workers. Another reason for a switch to smaller-sized units might be the tax exemption provided in the pre-GST era and the current post-GST era.

The present study captures valuable data on the profits of the *bidi* manufacturing industry. A sizeable increase in profits of the registered *bidi* manufacturing industry from 2005–2006 to 2010–2011 shows that only owners of these industries seem to be benefiting from manufacturing this product in the country, whereas *bidi* workers, consumers and the government appear to be at the losing end. The massive increase in profits between 2005–2006 to 2010–2011 might be attributed to a fall in real tax rate during these phases along with a reduction in labour cost. Since taxes on cigarette and *bidis* are on per thousand sticks (specific tax) the real tax rate falls over time if there is no annual revision of tax rate given the rate of inflation. The reduction in labour costs is also an important factor and this is clearly demonstrated in the analyses which showed that the actual wage rate declined over time especially with wages of *bidi* workers having seen a steep reduction from 2000–2001 to 2010–2011. Along with the increase in industry profits over this period, we found a marginal reduction in the total number of employees in the *bidi* industry. There was also no change in the guidelines for number of work hours (9 hours per day; 48 hours per week) for the *bidi* industry workers as specified by the Beedi And Cigar Workers (Conditions of Employment) Act, 1966 over this period (21). Hence, it is unlikely that reduction in the number of work hours for the *bidi* industry workers could have contributed to the wage decline.

The study highlights that on an average in 2010–2011, a *bidi* worker earned only about 17% of the wage of an average worker in the registered manufacturing sector. Thus, millions of *bidi* workers are employed in a non-remunerative job and condemned to poverty in comparison to working in other sectors in the manufacturing industry. Furthermore, with increasing contractual employment, labour cost due to lesser expenditure on social security is also likely to decline.

The study further highlights the exploitation of women as *bidi* workers. Female workers annually earned INR 8,000 (USD 178) less than males, which further reduced to INR 7,000 (USD 155.7) in 2005–2006. This disparity went down even more in 2010–2011 compared to previous levels to show the prevalence of a wide wage gap. This implies that the *bidi* industry is not empowering women economically, but rather exploiting existing social norms to pay unequal wages. Our findings are consistent with other studies which have suggested that women and children specially the girls perform better in bidi rolling, ironically, men earn more and their wages are higher in comparison to women (22), resulting in the exploitation of women who are actually bearing the dual workload. These low wages to women have an impact on the household economy especially in case of the household where women are the bread earners of the family and are principally involved only in bidi rolling process (7).

It is evident that the industry tends to hide its profits, arguing about employment and vulnerability of those hailing from a low socio-economic background and who happen to be engaged in the industry. While it may increase government tax revenue, the *bidi* industry may still find labour exploitative tactics. Therefore, it is imperative that the government focuses on formulating policies that aim to relocate workers employed in the *bidi* industry to alternate sectors which provide better job quality with higher wages, along with social security and employment benefits. This can be done under flagship schemes of the Government of India, such as the Skill Development Initiative Scheme under the Ministry of Skill Development & Entrepreneurship (23) and Ministry of Rural Development schemes like Deen Dayal Upadhyaya Grameen Kaushalya Yojana (24). The Ministry of Labour and Employment in collaboration with National Skill Development Corporation is providing skill-based training to *bidi* rollers and their dependents, to assist them to shift to more remunerative and healthy alternative sources of livelihood. However, there is a need to scale up the intervention, in view of the huge number of bidi rollers in India who are at risk and facing occupational hazards. The intervention is in line with the guidelines framed under Articles 17−18 of the WHO Framework Convention on Tobacco Control (25). Till such time that the Ministry of Labour and Employment is implementing alternate livelihood schemes, the unregistered sector may be regulated to shift to the registered sector for better accountability and enforcement of legislation, policies, taxation and programmes and enhanced welfare measures for the bidi workers.

## Strength and Limitations

In the present study, nationally representative data sets were used to study the trends in employment, profits and wages over the years. We also studied the profits earned by the bidi industry in relation to wages earned. This gives a clearer picture of the exploitation of the bidi workers in the form of low wages and income inequality.

Few limitations are that all the estimates in the present study are for years (2000–2001, 2005–2006, 2010–2011), however, profit estimates are for the year (2005–2006 and 2010–2011) only. Due to data issue for year 2000–2001, profit estimates were not calculated for this year. Even with two data points (2005–2006 – 2010–2011), it is evident that bidi industry earned huge profits over this period. Moreover, wages and profit calculations are for the registered sector, which constitutes only a small proportion of the bidi industry sector in India. However, for the unregistered sector, it was not possible to distinguish between the earnings made by the bidi enterprise and working owner's income. The profit estimations were not directly comparable between the year 2005–2006 and 2010–2011 hence they were adjusted for inflation using the Wholesale Price Index [WPI] (MP) base: 2004–2005 = 100. This was done to ensure the comparability across years. Also, data set used in this study provides enterprises unit wise data where the education level of each worker has not been mentioned. Moreover, this was an indicative study based on descriptive statistics. We have not conducted analysis to assess the determinants contributing to the increase in profit-GVA ratio or wage inequality. In this paper, we tried to highlight that share of profit in GVA is increasing over the years, and in the later stage we demonstrate falling wage rates of workers in the Indian Bidi industry, to indicate the possible labour exploitations taking place in this industry. Factors contributing to increase in profit-GVA ratio and wage inequality can be assessed through further research.

## Data Availability Statement

The data analyzed in this study is subject to the following licenses/restrictions: Data sets are not available in public domain but can be purchased on request from National Sample Survey Office (NSSO) and the Central Statistics Office (CSO), Government of India. Requests to access these datasets should be directed to http://www.mospi.gov.in/nsso; https://www.cso.ie/en/index.html.

## Ethics Statement

Ethical approval for this study and written informed consent from the participants of the study were not required in accordance with local legislation and national guidelines, as the study comprised of analysing secondary anonymous datasets.

## Author Contributions

MA and PD were involved in the overall design and analysis for this report. While AB conducted an analysis under the guidance of PD. MA led and supervised the entire study. SB provided critical inputs to the interpretation of data and framing of discussions on public health and policy implications of results. DB drafted the manuscript. GN provided critical inputs of the important intellectual content on the interpretation of results and the introduction. PS, VM, and FT provided critical technical inputs on the manuscript and revised the manuscript critically for intellectual content. All authors have read the manuscript and approve of its contents.

## Conflict of Interest

The authors declare that the research was conducted in the absence of any commercial or financial relationships that could be construed as a potential conflict of interest. The handling editor declared a past collaboration with one of the authors SB.

## References

[1] Ministry of Health and Family Welfare Tobacco Control in India. (2004). Available online at: https://main.mohfw.gov.in/sites/default/files/4898484716Report%20on%20Tobacco%20Control%20in%20India.pdf (accessed April 27, 2020).

[2] Tata Institute of Social Sciences Ministry of Health and Family Welfare Government of India Global Adult Tobacco Survey GATS 2 India 2016-17, (2018). Available online at: https://www.who.int/tobacco/surveillance/survey/gats/GATS_India_2016-17_FactSheet.pdf (accessed April 27, 2020).

[3] Ministry of Health and Family Welfare Government of India Global Adult Tobacco Survey 2009-2010. GATS. 2010 1-330. Available online at: https://www.who.int/tobacco/surveillance/survey/gats/gats_india_report.pdf (accessed April 27, 2020).

[4] Lal PG Wilson NC The Perverse Economics of the Bidi and Tendu Trade. Economic and Political Weekly. (2012). Available online at: https://www.researchgate.net/publication/262842790_The_Perverse_Economics_of_the_Bidi_and_Tendu_Trade (accessed April 27, 2020).

[5] Yasmin S Afroz B Hyat B D'Souza D Occupational health hazards in women beedi rollers in bihar, india. Bull Environ Contam Toxicol. (2010) 85:87–91. 10.1007/s00128-010-0037-6 20512312

[6] Health Mangasuli P Utilization pattern of social welfare schemes among women beedi workers in comparison with non-beedi workers. Int J Community Med. (2016) 3:3266–70. 10.18203/2394-6040.ijcmph20163948

[7] Madheswaran S Rajasekhar D Gayathiri Devi KG Production relations, employment and wages: a study of Beedi industry in Karnataka. Indian J Labour Econ. (2006) 49:643–60.

[8] John P Beedi Industry and Welfare of Workers in India Review of Policies and Literature. Available online at: http://www.chsj.org/uploads/1/0/2/1/10215849/policy_review.pdf (accessed January 25, 2020).

[9] Business Standard Pvt Ltd GST Council Decides to Exempt Businesses With Turnover of Rs 40 Lakh. (2019). Available online at: https://www.business-standard.com/article/news-ani/gst-council-decides-to-exempt-businesses-with-turnover-of-rs-40-lakh-119011000751_1.html (accessed April 27, 2020).

[10] Rustagi P Srivastave P Bhardwaj P Saha M Vyas A Shree M Survey of Studies on Beedi Industry With Special Emphasis on Women and Child Labour. (2001) 1–18.

[11] Nandi A Ashok A Guindon GE Chaloupka FJ Jha P. Estimates of the economic contributions of the bidi manufacturing industry in India. Tob Control. (2015) 24:369–75. 10.1136/tobaccocontrol-2013-051404 24789606

[12] Statistics MOF Office CS Annual Survey of Industries. Statistics (Ber) (2009). Available online at: http://www.csoisw.gov.in/cms/en/1023-annual-survey-of-industries.aspx (accessed April 27, 2020).

[13] National Sample Survey Organisation Ministry of Statistics and Programme Implementation Government of India Unorganised Manufacturing Sector in India 2000 - 2001 Employment, Assets and Borrowing. NSS 56th round (July 2000 – June 2001). Available online at: http://mospi.nic.in/sites/default/files/publication_reports/479_final.pdf (accessed April 27, 2020).

[14] National Sample Survey Organisation Unorganised Manufacturing Sector in India : Input, Output, and Value Added : NSS 62nd Round, July 2005-June 2006. Available online at: http://mospi.nic.in/sites/default/files/publication_reports/526_final.pdf (accessed April 27, 2020).

[15] National Sample Survey Office Ministry of Statistics and Programme Statistcs and Programme Implementation(MOSPI) Government of India Unorganised Manufacturing Sector in India Its Size, Employment and Some Key Estimates. NSS 51^st^ round: July 1994 - June 1995. Available online at: http://mospi.nic.in/sites/default/files/publication_reports/433_final.pdf (accessed April 30, 2020).

[16] World Health Organization (WHO) WHO Report on the Global Tobacco Epidemic. (2008). Available online at: https://www.who.int/tobacco/mpower/gtcr_download/en/ (accessed April 27, 2020).

[17] Selvaraj S Srivastava S Karan A Rout SK Arora M Fiscal Policies and Tobacco Control in India. New Delhi: Cambridge University Press (2016).

[18] John RM Dauchy E Goodchild M. Estimated impact of the GST on tobacco products in India. Tob Control. (2019) 28:506–12. 10.1136/tobaccocontrol-2018-054479 30219796

[19] Draft National Health Policy 2001-I : Debt Payment and Devaluing Elements of Public Health Econ Polit Wkly. (2015) 50:7–8.

[20] Mohandas M Kumar PVP Impact of Co-operativisation on Working Conditions: Study of Beedi Industry in Kerala. Econ Polit Weekly 1992;27(26):1333.

[21] The Beedi And Cigar Workers (Conditions of Employment) Act 1966 Available at http://labour.bih.nic.in/acts/beedi-and-cigar-workers-act-1966.pdf (accessed August 27, 2020).

[22] Sharma K Changing the Terms of the Discourse : Gender, Equality and the Indian State. New Delhi: Pearson (2011). p. 437

[23] Press Information Bureau 3620 Beedi Workers Trained Under the Skill Development Programme. (2019). Available online at: https://pib.gov.in/Pressreleaseshare.aspx?PRID=1579735#:~:text=3620%20Beedi%20Workers%20Trained%20Under%20the%20Skill%20Development%20Programme&text=This%20Ministry%20has%20initiated%20a,beedi%20workers%20and%20their%20dependents (accessed September 24, 2020).

[24] National Informatics Centre (NIC) Deen Dayal Upadhyaya Grameen Kaushalya Yojana (DDU-GKY), MoRD, Goverment of India. (2018). Available online at: http://ddugky.gov.in/ (accessed April 27, 2020).

[25] World Health Organisation Framework Convention on Tobacco Control (2003) Available online at: https://www.who.int/fctc/text_download/en/ (accessed August 25, 2020).

